# What Medical Writing Means To Me

**DOI:** 10.4103/0973-1229.32161

**Published:** 2007

**Authors:** Elizabeth Wager

**Affiliations:** *Medical writer, editor and trainer. Member, Ethics committees, BMJ and WAME; Council member, COPE. liz@sideview.demon.co.uk*

**Keywords:** *European Medical Writers Association (EMWA) Guidelines for medical writers*, *ghost writing*, *good publication practice guidelines*, *ICMJE*, *IMRAD format*, *medical writing*, *trial registration*

## Abstract

This is a personal account based on many years experience as a medical writer. It considers aspects of medical writing with particular focus on the intellectual and ethical dilemmas it can raise. What makes medical writing both so interesting and so challenging is the fact that it often takes place at the border between different disciplines. For example, it straddles both science and art. Ethical issues also arise at the boundaries between academia and commerce. Until recently there have been few guidelines to help navigate such potentially dangerous territory. I have been privileged to be involved in developing two such guidelines and I describe how I hope the Good Publication Practice guidelines for pharmaceutical companies and the European Medical Writers Association guidelines may improve the practice of this fascinating profession, Medical Writing.

## Introduction

Medical writing, like the treatments it describes, can be used to improve health but also has the potential to harm. Rudyard Kipling (Kipling, 1923) wrote that ‘words are, of course, the most powerful drug used by mankind’, reminding us that writing has the power to change behaviours and attitudes. Medical writing therefore carries a heavy burden of responsibility.

However, working with a powerful, or potentially dangerous, substance can be exhilarating. Another factor that makes medical writing such an interesting area is that it inhabits a strange boundary zone between science and art. Medical writing, especially reporting clinical trial results must be factual and objective. Certain aspects can undoubtedly be improved by following checklists and guidelines (Plint *et al*, 2006). Yet formulaic papers that report results dispassionately tend to be dry and uninteresting, while good papers should be inspiring and persuasive. But if the persuasive elements are taken too far or the arguments are not properly grounded in the findings, the report becomes biased and potentially misleading. Writers therefore walk a tightrope along what has been termed ‘the rhetoric of research’ (Horton, 1995; Schriger, 2005). They need to understand both the underlying science and the expressive art and to know where one should stop and the other begin. For example, it is acceptable, indeed often desirable, to omit measurements that have no bearing on a study's main findings from a publication. Details such as patient height are often recorded in clinical trials but need not be reported in a paper. A good writer knows that omitting such detail will create a shorter, clearer paper. However, if a drug is found to stunt the growth of children, this finding becomes material and a good scientist must ensure that the phenomenon is clearly described.

## Border Zones, Balance and Judgement

The type of medical writing I have mostly been involved with also takes me to another interesting, and potentially risky, border zone between the worlds of commerce and academia. Writers, hired or employed by sponsors, work closely with academic investigators. Sometimes the role feels more like peacemaker or arbitrator than wordsmith. On top of this, when preparing articles for journals, the writer not only has to keep the sponsor and the named authors happy, but also the journal editor and reviewers. Journal requirements can be a useful ally when trying to explain to an over-enthusiastic product sponsor that, although his company's policy may be to use only a product's trade name (without the generic) and that the name must always appear in capitals, such practice is quite unacceptable to journals and may even be counterproductive since reviewers will regard the paper as thinly veiled marketing, even though it may describe sound science. Sometimes it is the corporate sponsors who want to rein back academic authors, because drug companies' marketing activities are usually closely regulated and they know that they cannot use words such as ‘safe’ when promoting a drug. Once again, the medical writer may be caught in the midst of these discussions, acting as go-between.

Even when writing does not cross the sometimes war-torn border between science and industry, it always involves questions of balance and judgement. The Chinese scholar Lu Ji (who was born in 261 AD) wrote ‘Writing is a struggle between presence and absence’ and taught writers to ‘weigh each word on a scale; use a measuring cord to make your cuts’ (Barnstone and Ping, 1996). Had he lived today, I am sure he would have had no problems expressing himself clearly within the tight constraints of abstract or journal word limits.

Writers and critics of literature place great emphasis on style and format. Yet these are often ignored (or constrained) in scientific writing. The traditional IMRAD format of a medical paper (comprising Introduction, Methods, Results and Discussion) has evolved as a useful workhorse but may not be the best way to present findings to every audience and should not be considered sacred. Electronic publishing and the spread of the internet have opened exciting possibilities for communicating medical research but they have not been fully explored, let alone exploited (Wager, 2006a). The structure of scientific papers has remained virtually unchanged in the past 50 years (Smith, 2004) and many journals have simply converted their conventional pages from print into pixels without exploring the new possibilities.

## Clarity, Completeness and the Writer's Art

One of the paradoxes of communication and a challenge to anybody reporting research, is that powerful communication requires a simple message, yet science reporting is based on a wealth of data and complex ideas. The writer must strive for both clarity and completeness. Scientists often complain that journalists over-simplify their message, but sometimes we need to learn from the journalists' skills in producing readable and reader-centred writing. One of the potential benefits of electronic publishing is that it allows readers to get as much or as little detail as they choose and there are almost no space constraints. We therefore do not have to limit ourselves to the traditional 2000-5000 word article as the sole means to communicate our data. It is now possible to make raw data available via data banks (Sim and Detmer, 2005) and then provide templates or filters that present different levels of detail. Thus, one could generate a high level report suitable for regulators or people preparing systematic reviews and also a less detailed version for busy clinicians plus a range of presentations for patients without compromising the integrity of the data.

The writers' art is revealed when one considers the question: ‘what is the best way to present clinical research findings?’ One could argue that the most ‘honest’ method, allowing no over-interpretation or ‘spin’, would be to present raw data. Yet few would suggest that this is the ‘best’ way of communicating findings. Most clinicians, let alone patients, lack the statistical skills (or the time) to analyse data. So most people agree that it makes sense to present analysed data. Following the idea of presenting findings in the most objective way possible, we might propose posting full trial reports (of the type prepared for regulatory authorities) onto websites. But such reports often run to hundreds of pages and are composed of dense tables, which are daunting and uninformative for most readers and take many hours to work through. Such a solution (while it might reduce biased reporting) would surely not satisfy busy healthcare professionals. So we might agree to distil the data into something readable and, immediately, we create a role for somebody who must decide what to include and what to omit. Which parts can be grouped together? Which findings should be highlighted? These questions call for judgement as well as good communication skills.

## The question of context

Then there is the question of context. Readers cannot understand the findings of a single study in isolation; they must be interpreted within the context of what is already known about the subject. So we add summaries of previous work and, once again, take important judgements about what to include and what to omit. When reporting, it is also important to explain the strengths and weaknesses of the trial design and analysis methods. If we accept that most readers are not equipped to analyse the data themselves they need a trustworthy and competent guide to explain how this has been done. Ideally, the interpreter will provide the reader with enough information to decide whether the conclusions are valid but all this adds to the subjectivity of the reporting.

Having argued that we should not consider traditional journal articles as sacrosanct, this route of argument can seem to develop into a plea in favour of the IMRAD paper, which presents the research rationale, describes the methods, states the findings and puts them into context. But conventional papers published in medical journals often omit important details (Ioannidis and Lau, 2001; Chan *et al*, 2004) while remaining over-detailed and hard to read for non-specialists (Roberts, 1994). While the 3000-5000 word IMRAD article may represent a happy medium it is by no means perfect and we should not ignore the possibilities of presenting data in alternative forms. For example, results can be posted on websites with links to trial registers, thus linking the findings with details of the study methodology published before the trial started. Access to information about the study design can help readers detect whether all outcomes have been fully and responsibly reported. An even more radical step is to present results in an analysable databank (Sim and Detmer, 2005).

In this era of evidence-based everything, I hope research funders will focus attention on the effects of different reporting methods. By testing different methods on their target audiences, I believe we could greatly improve the efficacy of our communication. Yet surprisingly little is known about the most effective ways of reporting trials and, in particular, about the efficacy (or otherwise) of the established methods of quality control such as peer review and technical editing which have evolved to improve the process (Jefferson *et al*, 2002; Wager and Middleton, 2002). This is not to say that they do not work, simply that we do not have strong evidence that they do work. I hope research funders might begin to realise that, unless results are disseminated, they cannot be implemented; therefore research into methods of dissemination and quality control is essential.

## Good Publication Practice for Pharmaceutical Companies

Returning to the question of the harms that may be caused by irresponsible medical writing, one of the most satisfying aspects of my work has been developing guidelines to raise professional standards. The first was Good Publication Practice for pharmaceutical companies (GPP) (Wager *et al*, 2003). As the name suggests, GPP is aimed at improving the behaviour of drug companies in reporting trials. It was the first set of guidelines to establish clear roles for medical writers working with investigators and sponsors. For example, it recommends that first drafts of publications should not be prepared until the writer has consulted with the named authors and recommends that all authors should be given a chance to comment on an outline before the first draft is written. It also encourages writers to keep in close contact with named authors throughout the development process, but reminds the named authors that they retain final responsibility for any publication bearing their name.

The GPP guidelines call on companies to endeavour to publish results of all their clinical trials. It met with considerable resistance when first proposed in the late 1990s. However, since then, real progress has been made with trial registration and this initiative was given a massive boost when major journals edited by members of the International Committee of Medical Journal Editors (ICMJE) announced that they would not publish trials unless they had been properly registered at their outset (De Angelis *et al*, 2004, 2005). One of the major aims of trial registration is to eradicate the twin problems of non-publication and redundant publication, which, together, create publication bias. This systematic bias means that unfavourable or non-significant results are less likely to be published while favourable or significant findings may be published (and, of greater concern, incorporated into meta-analyses) more than once (Tramér *et al*, 1997). However, on its own, trial registration cannot prevent such behaviour (which has, rightly in my view, been described as a form of scientific misconduct (Chalmers, 1990). In order to do this, it must be coupled with a commitment to report not just the design but also the results of all trials. However, for any guideline to be enforceable it must be unambiguous and there is currently an urgent need to define exactly what we mean by publication (Wager, 2006a).

## EMWA Code of Practice for Medical Writers

Building on guidelines for companies, I also helped to develop a more detailed code of practice for individual medical writers under the auspices of the European Medical Writers Association (EMWA) (Jacobs and Wager, 2005). This was needed to further clarify the appropriate role for medical writers in developing publications and to provide guidance on difficult aspects such as authorship and acknowledgement which were not covered in detail in GPP. This is an area in which medical writing clearly differs from other literary endeavours. In most cases (except perhaps, ghosted celebrity autobiographies) the authorship of literary works is uncontroversial. Novelists, poets and columnists make their living by having original ideas and effective ways of expressing them. But scientific research usually involves complex collaborations and teams of people. Thus, responsibility for designing the study, carrying it out, analysing the data and writing the report are often shared among several individuals. Many branches of medical research have become so specialized that a large multi-skilled team is required to carry out a clinical trial and it is recognised that team members bring different skills to the endeavour. The allocation of authorship for scientific papers can therefore be controversial. Even the most widely respected guidelines on authorship have had to be revised to reflect this and now demand that most authors take responsibility for only a portion of the work rather than the entire study (ICMJE, 2007). This makes sense, since one would not expect a pathologist to be able to defend the statistical methods used in a trial to the same extent as the statistician or vice versa; but it increases the gap between generally agreed concepts of literary authorship and standards of recognition in the scientific literature. Recognition of the multi-disciplinary team structure of much clinical research justifies, in my mind at least, the involvement of professional medical writers who may not necessarily have been involved in other aspects of the research. If it is acceptable (in fact, desirable) to enlist the services of a professional statistician to advise about study design, data analysis, etc. why, then, should it be unethical to hire a professional writer or somebody with skill and experience in developing publications, to help with these aspects, so long as their role and any potential competing interests are clearly described?

The problem, I believe, is not the involvement of writers per se, but lack of transparency when their role or funding is obscured. For this reason, both the GPP and EMWA guidelines highlight the importance of proper acknowledgement. Ghost writers lose their menacing spookiness if they are clearly named and many journals are now stepping up their efforts to ensure that this happens (Groves, 2007). Over the last decade I have experienced a remarkable change from most journal editors being openly hostile to medical writers to many realising that writers may actually be helpful but that problems arise when their role is not properly acknowledged. However, academic authors may still be reluctant to admit that they received professional help in preparing a paper, so they may collude with sponsors who wish to play down their involvement by hiding the contribution of a paid writer. Further work educating all parties and promoting good practice is needed to shift the culture away from shameful hauntings to admirable transparency. The simple remedy of journals listing individuals' contributions rather than simply their names and affiliations on a by-line will help to accelerate this process (Wager, 2006b).

## Concluding Remarks

What does the future hold for medical writing? I believe the ever-growing body of medical data and opportunities provided by electronic publication offer exciting possibilities for harnessing our creative talents and devising new and more effective methods of communicating the results of clinical research. There is a hunger for such information from healthcare professionals, legislators, regulators, patients and carers, yet much of it remains inaccessible. It is a fallacy to believe that science can be reported completely dispassionately and without some form of interpretation, so writers combining a robust understanding of the science and the skills to communicate messages clearly will always be in demand. However, the powerful tool of writing can be subverted and used irresponsibly, so we need guidelines to encourage best practice.

I believe we also need more research so that we understand the complex psycho-social processes involved in reporting and reading reports of scientific research. My vision for the future is of evidence-based practice firmly grounded in ethical principles, promoted and taught by professional experts. Such training should be integrated within medical (and allied health professional) education. For, while the skills of professional writers should be nurtured and respected, we should not forget that the majority of medical writing will continue to be done by non-professionals. All writers can benefit from training, as was recognised over 300 years ago by Alexander Pope (Pope, 1955), who wrote:

‘*True ease in writing comes from art, not chance/*

As those move easiest who have learned to dance.’

### Take home message

Medical writing can enhance scientific communication and the dissemination of research findings but can also be used irresponsibly. Professional writers, journal editors and academic investigators need to understand both the opportunities and the risks and work together to enforce good practice and high standards. Training in both the technical and ethical aspects of the craft should be encouraged for everybody who contributes to the medical literature.

## Questions that this paper raises

- How can we exploit the possibilities of electronic media and the Internet to improve the quality of reporting medical research and making information accessible for different audiences?
- There is little evidence that peer review is effective in raising the quality of medical articles - can we devise better methods of quality control?
- Hidden conflicts of interest threaten the integrity of publications - how can journal editors ensure that the involvement of medical writers and their funding sources are always acknowledged?
- In the past, some academics have colluded with commercial sponsors by failing to acknowledge the involvement of medical writers. How can we achieve a change of culture so that involving a professional writer is viewed as good practice (similar to involving a statistician) rather than something to be ashamed about?
- Should medical writing be more formally regulated, e.g. with a professional governing body, entrance qualifications, etc.?

## About the Author

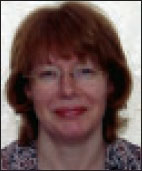



Liz Wager is a freelance medical writer, editor and trainer based in Princes Risborough, England. Before setting up her own company, Sideview (in 2001), she worked for Janssen Cilag, GlaxoWellcome and Blackwell Scientific Publications. She has a particular interest in publication ethics and is a member of the ethics committees for the BMJ and WAME (World Association of Medical Editors) and a council member of COPE (the Committee On Publication Ethics). She has run workshops on writing, publication strategy and publication ethics on five continents. She has also written a book on publication strategy and co-authored one on peer review. She enjoys running (occasionally managing a half marathon) and spoiling her four cats.
